# Targeting Tyrosine Phosphatases by 3-Bromopyruvate Overcomes Hyperactivation of Platelets from Gastrointestinal Cancer Patients

**DOI:** 10.3390/jcm8070936

**Published:** 2019-06-28

**Authors:** Alessandra V. S. Faria, Sheila S. Andrade, Agnes N. Reijm, Manon C. W. Spaander, Moniek P. M. de Maat, Maikel P. Peppelenbosch, Carmen V. Ferreira-Halder, Gwenny M. Fuhler

**Affiliations:** 1 Department of Gastroenterology and Hepatology, Erasmus University Medical Center Rotterdam, NL-3000 CA Rotterdam, The Netherlands; 2 Department of Biochemistry and Tissue Biology, University of Campinas, UNICAMP, Campinas 13083-862, SP, Brazil; 3 PlateInnove Biotechnology, 13414-018 Piracicaba, SP, Brazil; 4 Department of Hematology, Erasmus University Medical Center Rotterdam, NL-3000 CA Rotterdam, The Netherlands

**Keywords:** platelet function, gastrointestinal cancer, venous thromboembolism, tyrosine phosphatases, LMWPTP, ACP1, PTP1B

## Abstract

Venous thromboembolism (VTE) is one of the most common causes of cancer related mortality. It has been speculated that hypercoagulation in cancer patients is triggered by direct or indirect contact of platelets with tumor cells, however the underlying molecular mechanisms involved are currently unknown. Unraveling these mechanisms may provide potential avenues for preventing platelet-tumor cell aggregation. Here, we investigated the role of protein tyrosine phosphatases in the functionality of platelets in both healthy individuals and patients with gastrointestinal cancer, and determined their use as a target to inhibit platelet hyperactivity. This is the first study to demonstrate that platelet agonists selectively activate low molecular weight protein tyrosine phosphatase (LMWPTP) and PTP1B, resulting in activation of Src, a tyrosine kinase known to contribute to several platelet functions. Furthermore, we demonstrate that these phosphatases are a target for 3-bromopyruvate (3-BP), a lactic acid analog currently investigated for its use in the treatment of various metabolic tumors. Our data indicate that 3-BP reduces Src activity, platelet aggregation, expression of platelet activation makers and platelet-tumor cell interaction. Thus, in addition to its anti-carcinogenic effects, 3-BP may also be effective in preventing platelet-tumor cell aggregationin cancer patients and therefore may reduce cancer mortality by limiting VTE in patients.

## 1. Introduction

In 2018, over 18 million new cases of cancer were diagnosed worldwide. Despite improvements in cancer treatment, mortality rates are still high [1]. One of the most common comorbidities of cancer is thrombosis. Overall, 20% of cancer patients experience a thrombotic event, and in patients with gastrointestinal (GI) cancers the risk of developing a venous thromboembolism (VTE) is particularly high [2,3]. The association between cancer and thromboembolism events is termed Trousseau Syndrome, after Armand Trousseau [4], who first described the high occurrence of superficial migratory thrombophlebitis [5]. Because thrombosis is a common comorbidity in cancer, treatment strategies have been devised to include the use of anti-thrombotic drugs, such as low molecular weight heparin (LMWH), aspirin and warfarin, for co-adjuvant therapy in cancer treatment [6]. However, these drugs decrease the overall thrombus formation, and as a consequence, side effects pose a challenge [7]. Finding new compounds that combat VTE as well as primary cancer cells could mean a step forward in cancer treatment. In cancer patients, platelets appear to be more easily activated by agonists as compared to platelets from healthy individuals, and this hyperactivity may relate to VTE risk. However, despite the high impact of VTE in cancer morbidity, it is as yet unclear how platelet phenotype and inherent function are modulated in patients with cancer [8,9,10,11]. Thus, elucidating the molecular mechanisms related to cancer-associated VTE remains crucial.

Platelets contain a vast array of proteins, such as membrane proteins (e.g., glycoprotein IIb/IIIa integrins, P-Selectin, CD36), adhesive proteins (e.g., von Willebrand factor, fibrinogen, vitronectin), growth factors (PDGF, VEGF, EGF, TGF-B, and others) and clotting factors (V, IX, and XIII) [11]. Upon tissue damage, soluble von Willebrand factor binds to the exposed collagen and subsequently tethers platelets by binding to their glycoprotein Ib receptors (GPIbR) [12], thereby providing a scaffold for the generation of thrombin and formation of fibrin fibers. Coagulation in thrombosis and hemostasis is well described [13]. One important emerging regulator of collagen receptor and integrin-mediated platelet function is the Src family of kinases [14,15], although how these kinases themselves are regulated in platelets remains relatively unclear [16]. We and others have previously demonstrated that in hematopoietic and GI tumor cells, modulation of Src is dependent on protein tyrosine phosphatase activity, and inhibition of these phosphatases attenuates Src-dependent cancer cell growth and metastasis [17]. One potential modulator of this intracellular signaling pathway is the small molecule 3-bromopyruvate (3-BP), which is known to kill metabolically active tumor cells through inhibition of glycolysis. The use of 3-BP for cancer treatment has been advocated [18,19]. Here, we investigate whether this compound may also hold promise for the prevention of platelet-tumor cell aggregation in cancer patients. We demonstrate for the first time that activity of Low Molecular Weight Protein Tyrosine Phosphatase (LMWPTP) as well as Protein Tyrosine Phosphatase 1B (PTP1B) in platelets is selectively modulated by platelet agonists. We show that these phosphatases are a target for 3-BP, which also inhibits Src activity in platelets. Furthermore, 3-BP reduces collagen-induced aggregation and activation of platelets from both healthy controls and GI cancer patients, demonstrating the potential anti-thrombotic effect of this compound. Thus, 3-BP-like molecules may hold promise as an anti-tumor agent which simultaneously prevents platelet-tumor cell aggregation. 

## 2. Material and Methods

### 2.1. Antibodies and Reagents

Antibodies were purchased from Santa Cruz Biotechnology (Dallas, TX, USA), Cell Signaling Technology (Danvers, MA, USA), SignalWay (College Park, MD, USA). For details, see Supplemental File Table S1. Reagents were purchased from Sigma Aldrich, Santa Cruz, Merck Millipore, Chronolog. For details, see Supplemental File Table S2.

### 2.2. Cell Culture

HT29, HCT116 and Caco-2 cells were obtained from ATCC (American Type Culture Collection, VA, USA) and routinely maintained in Dulbeco’s Modified Eagles Medium (DMEM, Lonza, Basel, Switzerland) supplemented with 100 U/mL penicillin, 100 mg/mL streptomycin (Life technologies, Bleiswijk, NL) and 10% Fetal Calf serum (FCS, Sigma-Aldrich, St. Louis, MO, USA) at 37 °C under a 5% CO_2_ humidified atmosphere. See Supplemental File Table S3 for characteristics of these lines.

### 2.3. Platelet Preparation 

After signed informed consent was obtained (Ethical committee Project NL66029.078.18 approved by Erasmus MC medical and ethical committee), venous blood from healthy donors (*n* = 19) and gastrointestinal cancer patients (*n* = 3) was collected into conical plastic tubes containing 3.8% trisodium citrate 1:10 (*v/v*). Whole blood was centrifuged at 1500 rpm, 10 min, 22 °C, and Platelet-Rich Plasma (PRP) was collected. For specific analysis NaCl (0.9%) was used to wash the platelets as previously described before [20]. All experiments were performed using 200–300 × 10^3^ platelets/µL. Due to logistical restraints, not all experiments were performed on all donors. The number of times an experiment was performed is indicated in the figure legends.

### 2.4. Patient Information 

Blood was obtained at diagnosis from three patients suffering from malignant esophageal neoplasia. The mean age was 73 ± 10 years, and two of them were male. Two patients used salbutamol, two patients took gastric pH modulators (Esomeprazol, Famotidine), and two patients used antidiabetics (hydrochlorothiazide). One patient took paracetamol, and one patient took metoclopramide as well as beclomethasone. None of these drugs were described to have an antiplatelet effect according to Chronolog (Chronolog Corp., Havertown, PA, USA). All cancer patients were gender-matched to a healthy control.

### 2.5. Platelet Aggregation Assay by Light Transmission

A 500 µL aliquot of PRP was placed in an aggregometer cuvette and incubated at 37 °C for 5 min in the presence or absence of compounds (100 µM 3-bromopyruvate, 10 µM CinnGEL, 100 µM NSC87887). Subsequently, the agonist collagen (2 µg/mL) was added to the samples. An aggregation curve was recorded for 10 min after the addition of agonist. Light transmission changes (an indicator of aggregation) were monitored with an aggregometer (Chrono-Log Corp.) under shear stress conditions by stirring at 1200 rpm following the method described before [21]. Quality controls of platelets were assessed by aggregation response at the beginning and end of experiments.

### 2.6. Platelet Activation Assay

Washed platelets were incubated with a final concentration of 100 µM 3-bromopyruvate, 10 µM CinnGEL, or 100 µM NSC87887, for 60 min at room temperature, followed by stimulation of platelets with 2 µg/mL Collagen or 1.25 mg/mL Ristocetin for 10 min. After treatment, samples were incubated with antibodies CD41b (92800/040408 M1674); CD42b (65117/151106 M1729); CD62 (AK4) (304910-B239360 Becton, Dickinson and Company, Franklin Lakes, NJ, USA) for 15 min and data was acquired using a MACSQuant^®^ Analyzer 10. Data analysis was performed with FlowJo, LLC v10 (Becton, Dickinson and Company, Franklin Lakes, NJ, USA). 

### 2.7. Platelet-Cancer Cells Interaction Assays

For co-culture experiments, colorectal cancer cells (HCT116, Caco-2 and HT29) (1.0 × 10^4^ cells/cm^2^) were plated in 24-well plates for 24 h. After that, cells were washed with PBS, and PRP was added to each well for 6h, together with either collagen (2 µg/mL), 3-BP (100 µM), or no agonists. After co-culture, the cells were either imaged by microscopy (Nikon), and the platelet-tumor cell aggregates were counted using a 10× magnification, at the well center quadrant, or platelets were harvested and analyzed by western blot as described before [22]. 

For aggregation assays in the presence of cancer cells, cancer cell lines were detached with trypsin-EDTA and washed several times with NaCl 0.9% to remove the excess of trypsin-EDTA. 500 uL of PRP was incubated with tumor cells (1.5 × 10^4^ cells/test in NaCl 0.9%)—(protocol described before [23] with some modifications) at 37 °C for 5 min in the presence or absence of 100 µM 3-BP. Subsequently, the agonist collagen (2 µg/mL) was added to the samples. An aggregation curve was recorded for 10 min after the addition of agonist. Light transmission changes (an indicator of aggregation) were monitored with an aggregometer (Chrono-Log Corp.) following the method described before [21]. Quality controls of platelets were assessed by aggregation response at the beginning and end of experiments.

### 2.8. Western Blot 

Two different platelet treatments were performed: (A) washed platelets (20,000,000-30,000,000) were incubated for 5 min in the absence or presence of compounds (final concentration: 100µM 3-BP, 10 µM CinnGEL, 100 µM NSC87887). Subsequently, platelet agonist collagen (2 µg/mL) was added to the samples and after 10 min, the platelets were collected, washed and lysed as described below; (B) Platelets collected from co-cultures with colorectal cancer cells were washed with NaCl 0.9% and lysed in 2× concentrated Laemmli buffer (100 mM Tris-HCl [pH 6.8], 200 mM dithiothreitol, 4% SDS, 0.1% bromophenol blue and 20% glycerol) and samples were boiled for 10 min. Cell extracts were resolved by SDS-PAGE (sodium dodecyl sulfate-polyacrylamide gel electrophoresis) and transferred to polyvinylidene difluoride membranes (Merck chemicals BV, Darmstadt, Germany). Membranes were blocked in 50% odyssey blocking buffer (LI-COR Biosciences, Lincoln, NE, USA) in TBS and incubated overnight at 4 °C with a primary antibody. After washing in TBS-T (TBS with 0.5% Tween 20), membranes were incubated with IRDye antibodies (LI-COR Biosciences, Lincoln, NE, USA) for 1 h. Detection was performed using Odyssey reader and analyzed using the manufacturer’s software. For the primary antibodies used, see Supplementary Table S1.

### 2.9. Immunoprecipitation Phosphatases

Platelets were treated with test compounds and subsequently stimulated with agonists as described above. Immunoprecipitation was performed for LMWPTP and PTP1B as described previously [24,25]. Briefly, cells were lysed with 100 μL lysis buffer (20 mM HEPES, pH7.4 with 2.5 mM MgCl_2_, 0.1 mM EDTA) on ice for 2 h. After clarifying by centrifugation and pre-clearing with uncoupled G-Sepharose beads (Thermo Fisher Scientific, Waltham, MA, USA), the platelet extracts were incubated overnight at 4 °C under rotation with antibodies against LMWPTP (Acp1) or PTP1B. G-Sepharose beads were added to lysate and incubated for 3 h at 4 °C. Samples were washed 3 times with acetate buffer (100 mM pH5.5) before performing phosphatase activity assays. 

### 2.10. Phosphatase Activity Assay

After immunoprecipitation (IP), the pellet was re-suspended in acetate buffer and the enzymatic activity was assessed as follows: reaction medium (100 μL) containing 100 mM acetate buffer, 5 mM p-nitrophenyl phosphate (PNPP) was added to the precipitated phosphatase. After 60 min at 37 °C and under agitation (600 rpm) the reaction was stopped by adding 100 μL 1M NaOH. The absorbance was measured at 405 nm (spectrophotometer-BioRad, Hercules, CA, USA), and results are indicated as optical density measured normalized for bead controls (OD).

The effect of 3-BP and CinnGEL on LMWPTP activity was examined after 10 min of pre-incubation with LMWPTP immunopreciptated as described above. Subsequently, the substrate was added to the reaction medium.

The effect of 3-BP and CinnGEL on PTP1B activity was examined after 10 min of pre-incubation with recombinant PTP1B (0010896, lot 04529). Subsequently, the substrate was added to the reaction medium.

### 2.11. MTT Assay

MTT assay was performed as described before [26]. Briefly, platelets were seeded into a 96-wells plate for 3 h (total volume per well 180 µL). 20 µL of MTT (Sigma Aldrich) solution (5 mg/mL in PBS) was added to each well. After incubating for 4 h at 37 °C, the plate was centrifuged 2500 rpm, 10 min, the MTT solution was removed and the formed formazan crystals were solubilized in 100 μL of ethanol. The absorbance was measured at λ = 585 nm with a microplate reader (BioRad).

### 2.12. Statistical Analysis

The data is represented by means ± SEM. Statistical analysis was performed using *t*-student (paired, 95% confidence intervals, two tailed) using GraphPad (version 5.0, GraphPad Inc, San Diego, CA, USA).

## 3. Results

### 3.1. Protein Tyrosine Phosphatases Are Selectively Activated by Classic Platelet Agonists in Healthy Blood Donors

**Figure 1:**
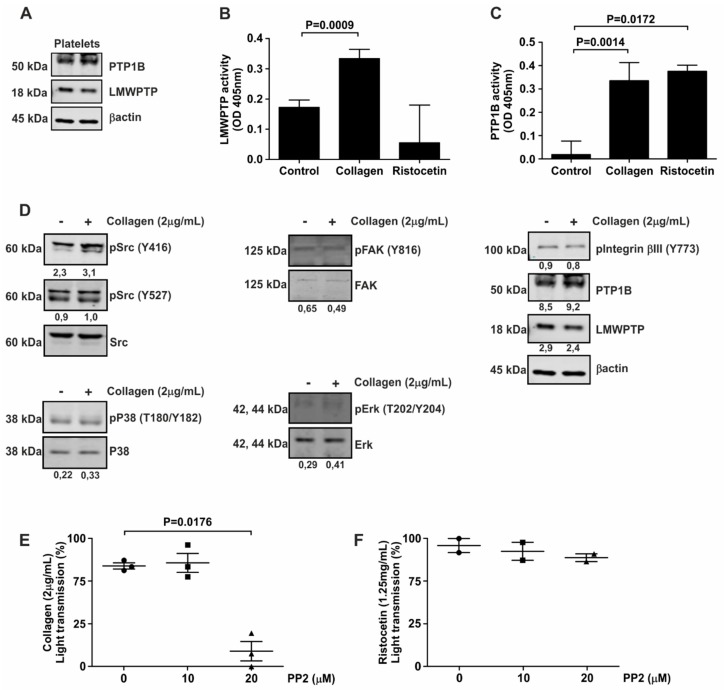
Platelets contain LMWPTP and PTP1B activity, which are selectively activated by platelet agonists. (**A**) Western blot analysis of platelets from two independent donors indicates protein expression of LMWPTP and PTP1B in these cells. (**B**,**C**) Platelets were stimulated with either collagen (2 µg/mL) or ristocetin (1.25 mg/mL) and LMWPTP (**B**) and PTP1B (**C**) were immunoprecipitated from the platelet lysates and subjected to phosphatase activity assay. Statistical analysis was performed using *t*-student (paired, 95% confidence intervals, two tailed) (*n* = 4). (**D**) Platelets were stimulated with collagen (2 μg/mL) and subjected to western blot analysis of the indicated (phospho-)proteins. β-actin served as a loading control. (−) Without collagen; (+) With collagen. The numbers under the blot indicate densitometry values corrected for loading controls. A representative blot of at least two independent experiments is shown. (**E**,**F**) Following pre-incubation with PP2 for 5 min, platelets were stimulated with either collagen (2 μg/mL) (E) or ristocetin (1.25 mg/mL) (**F**) and the aggregation was measured for 10 min. Each data point corresponds to an individual single experiment, indicated by: Circle—0 μM; Square—50 μM and Triangle—100 μM condition.

The role of kinases in platelet biology has received much more attention than phosphatases. We therefore set out to investigate the expression and activity of two tyrosine phosphatases known to be overexpressed in gastrointestinal cancer; LMWPTP and PTP1B. Our findings show that both of these phosphatases are expressed in human platelets (Figure 1A). Next, we investigated the activity of these phosphatases in the presence of either the physiological agonist collagen or the synthetic agonist ristocetin, both of which activate a robust platelet aggregation response (Supplemental Figure S1). As demonstrated in Figure 1B, constitutive activity of LMWPTP was present in platelets, which could be further increased by stimulation of cells with collagen (2 µg/mL), but not with ristocetin. In contrast, constitutive activity of PTP1B in platelets was lower, but drastically enhanced by treatment with either collagen or ristocetin (Figure 1C). These data suggest that PTP1B activity is a general response to platelet activation, whereas LMWPTP activity is dependent on the selective agonist used. As tyrosine phosphatase activity generally affects cellular protein phosphorylation levels, we next determined the phosphorylation status of several known targets of LMWPTP and PTP1B [24,25]. Constitutive phosphorylation of FAK, Integrinβ3 and p38 was present in platelets, but the most noticeable activation of signaling upon collagen stimulation was seen for the Src family kinases, as determined by their phosphorylation at tyrosine residue Y416 (Figure 1D). To confirm the importance of Src for platelet function, we performed aggregation assays in the presence of the selective Src family kinase inhibitor PP2. Interestingly, only collagen-stimulated aggregation was reduced in the presence of PP2 (Figure 1E, Figure S1-I), while ristocetin-induced aggregation was not (Figure 1F, Figure S1-I), suggesting that collagen activation of platelets in particular depends on Src signaling. 

### 3.2. Collagen-Induced Intracellular Signaling in Platelets from Healthy Donors is Inhibited by 3-BP

**Figure 2:**
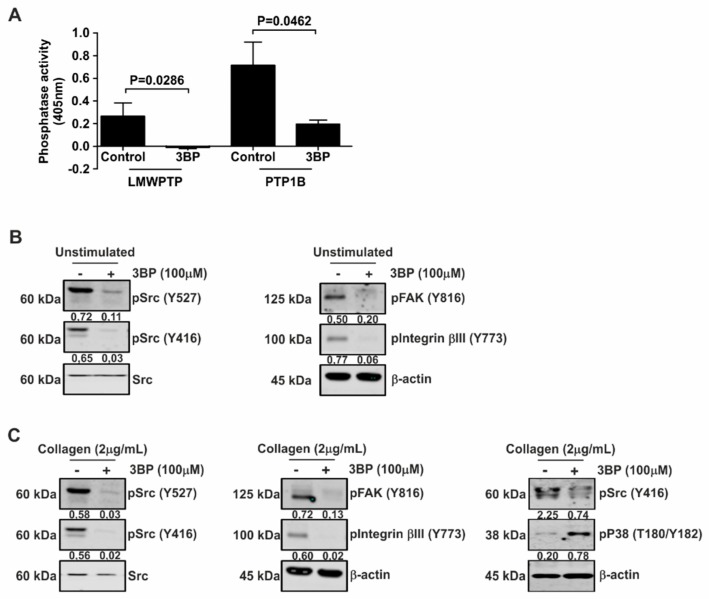
3-BP inhibits intracellular signaling in platelets. (**A**) Platelets were stimulated with collagen (2 μg/mL) and LMWPTP was immunoprecipitated. For PTP1B activity assay, active human recombinant protein was used. Following treatment with 3-BP, precipitates were subjected to phosphatase activity assays. Statistical analysis was performed using *t*-student (paired, 95% confidence intervals, two tailed) (*n* = 4). (**B**,**C**) Platelets were pretreated with 3-BP for 30 min and left either unstimulated (B) or were treated with collagen (**C**). Western blot analysis was performed for the indicated proteins, with β-actin serving as loading control. The numbers under the blot indicate densitometry values that were corrected for loading controls. A representative blot of at least two independent experiments is shown.

3-BP has been suggested as a promising antitumor agent by targeting a set of key metabolic enzymes, including kinases [27]. Therefore, we investigated the effect of this compound on cellular signaling in platelets. Interestingly, both collagen-induced LMWPTP activity and PTP1B activity were significantly reduced by 3-BP (Figure 2A). Furthermore, pretreatment of platelets with 3-BP drastically reduced both constitutive (Figure 2B) and collagen-stimulated levels (Figure 2C) of Src, FAK, and Integrinβ3 phosphorylation. However, the MAPK p38 was activated as demonstrated by an increase of the phosphorylation of T180 and Y182 residues (Figure 2C). Taken together, these data suggest that stimulation of platelets with collagen stimulates phosphatase activity and enhances Src activity, both of which are reduced by 3-BP treatment.

### 3.3. 3-BP Abrogates Collagen-Induced Platelet Aggregation

**Figure 3:**
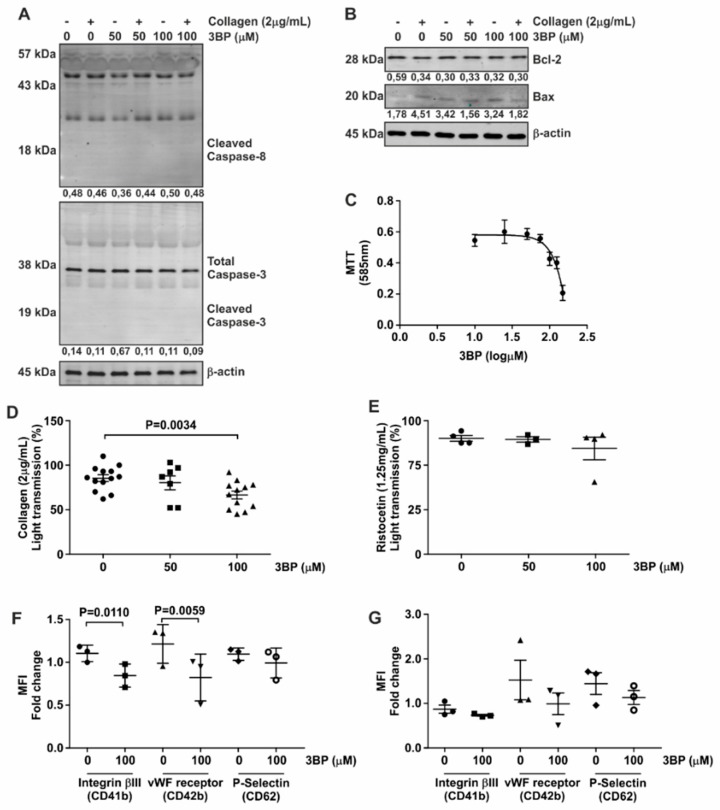
3-BP decrease affects platelet metabolic activity and function. (**A**,**B**) Platelets were subjected to 50 μM and 100 μM 3-BP for the indicated time points, and expression of Bcl2 and BAX (**A**) as well as caspase cleaving (**B**) were determined by Western blot analysis. β-actin served as loading control. The numbers under the blot indicate densitometry values corrected for loading controls. A representative blot of at least two independent experiments is shown. (**C**) Isolated platelets were subjected to the indicated concentrations of 3-BP, and cell metabolic activity was followed by MTT assay (mean ± SEM shown). (**D**,**E**). Aggregation assay using 3-BP as inhibitor of platelet function. Following preincubation with 3-BP (5 min) platelets were stimulated with either collagen (2 μg/mL) (D) or ristocetin (1.25 mg/mL) (**E**) and the aggregation was measured for 10 min. Circle indicates 0μM; Square indicates 50 μM and Triangle indicates 100μM condition. (**F**,**G**) Expression of platelet activation markers in the presence of 3-BP. Platelets were stimulated with either collagen (2 μg/mL) (F) or ristocetin (1.25 mg/mL) (**G**) and stained using CD41b-FITC, CD42b-PE and CD62-APC antibodies to detect surface expression of vWF-receptor, Integrinβ3 and P-Selectin, respectively. Statistical analysis was performed using *t*-student (paired, 95% confidence intervals, two tailed). Each data point (special shapes) corresponds to an individual experiment and indicates the data as: Circle—Integrinβ3 without 3BP, Square—Integrinβ3 with 3BP, Up-triangle—vWF-receptor without 3BP, Down-triangle—vWF-receptor with 3BP, Diamond—P-Selectin without 3BP, Hollow circle—P-Selectin with 3BP.

Based on the inhibitory effect of 3-BP on both kinases and phosphatases in platelets, we set out to investigate the functionality of these cells in the presence of this compound. We first confirmed that 3-BP was not cytotoxic to platelets, by demonstrating that neither caspase-3 nor caspase-8 integrity, both of which are cleaved upon apoptosis induction [28,29], were changed upon 3-BP treatment (Figure 3A). Furthermore, expression levels of pro-apoptotic BAX and the anti-apoptotic Bcl-2 proteins (Figure 3B) were unaffected by 3-BP treatment of platelets.

The glycolytic pathway has been described as an important mediator of platelet function [30,31,32]. 3-BP is able to inhibit enzymes from this metabolic pathway [32]. This was confirmed by our finding that 3-BP reduces tetrazolium formation, known to be dependent on cellular glucose metabolism, by platelets (Figure 3C) [33]. 

In light of the involvement of Src in collagen-mediated platelet aggregation and the inhibitory effect of 3-BP on Scr signaling, we next investigated whether 3-BP could disturb platelet activation and aggregation-specific events. As shown in Figure 3D and Figure S1-II, 3-BP at a concentration of 100 μM was able to inhibit platelet aggregation induced by collagen, which binds to integrin α1β2 and glycoprotein GpVI on the platelet surface [34,35]. In contrast, platelet aggregation induced by ristocetin and mediated via vWF binding to Gp1b receptors [36], was not reduced by pretreatment of platelets with 3-BP (Figure 3E, Figure S1-II). Activation of platelets was accompanied by increased expression of Integrinβ3, vWF receptor and P-Selectin on the cell surface, and 3-BP treatment of platelets significantly reduced collagen-induced expression of both Integrinβ3 and vWF receptor on these cells as determined by FACS analysis (Figure 3F), while the expression of these receptors in the presence of ristocetin was not affected (Figure 3G). Thus, we conclude that 3-BP induces selective inactivation of platelets, without causing wide-scale apoptosis.

### 3.4. Platelet Function Is Dependent on Specific Phosphatases

**Figure 4:**
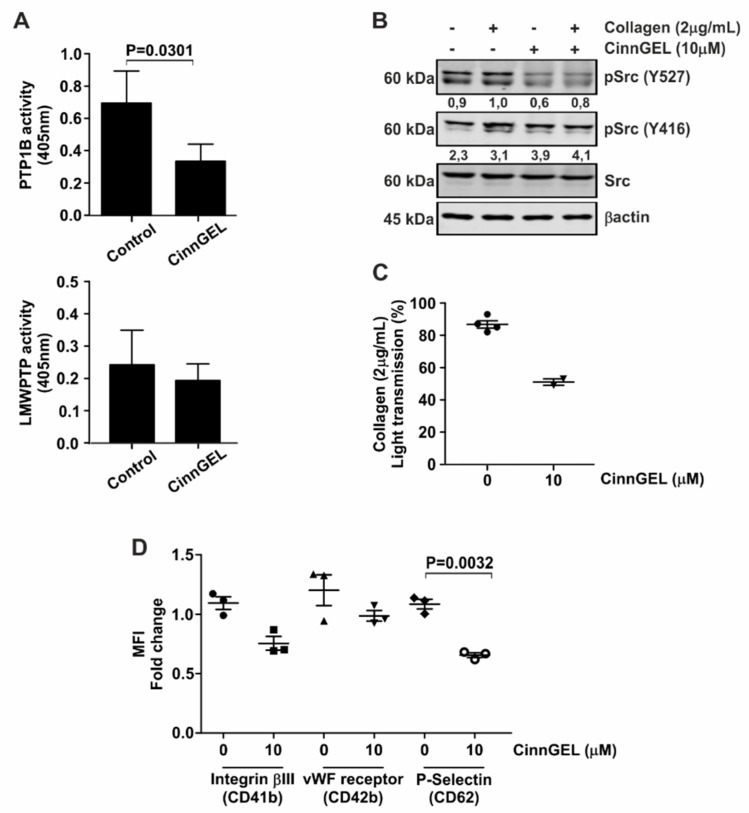
Platelet function is inhibited by selective PTP1B inhibition. (**A**) PTP1B activity assays were performed using active recombinant human protein. For LMWPTP activity assays, platelets were stimulated with collagen (2 μg/mL) and LMWPTP was immunoprecipitated. Following treatment with CinnGEL (PTP1B inhibitor), recombinant proteins and precipitates were subjected to phosphatase activity assays. (**B**) Platelets were pretreated with CinnGEL or left untreated for 5 min. Cells were subsequently left either unstimulated or were treated with collagen. Western blot analysis was performed for the indicated proteins, with β-actin serving as loading control. The numbers under the blot indicate densitometry values corrected for loading controls. A representative blot of at least two independent experiments is shown. (**C**) Aggregation assays were performed for collagen-stimulated platelets after pretreatment with CinnGEL. (**D**) Cell surface expression of vWF-receptor (CD41-FITC), Integrinβ3 (CD42-PE) and P-Selectin (CD62-APC) was investigated on collagen-stimulated platelets that were pre-treated with CinnGEL. Each data point (special shapes) corresponds to an individual experiment and indicates the data as: Circle—Integrinβ3 without 3BP, Square—Integrinβ3 with 3BP, Up-triangle—vWF-receptor without 3BP, Down-triangle—vWF-receptor with 3BP, Diamond—P-Selectin without 3BP, Hollow circle—P-Selectin with 3BP.

3-BP is not a specific inhibitor of PTPs. Therefore, to confirm the involvement of phosphatases in platelet activation, we employed the PTP1B inhibitor CinnGEL. As expected, CinnGEL specifically inhibits PTP1B (Figure 4A, upper panel), but not LMWPTP (Figure 4A, lower panel). Furthermore, inhibition of PTP1B was accompanied by a reduced activation of Src (Figure 4B). Investigation of platelet activation in the presence of CinnGEL demonstrated that platelet aggregation was significantly diminished upon PTP1B inhibition (Figure 4C). In addition, collagen-induced expression of platelet activation markers Integrinβ3, vWF receptor and P-Selectin were significantly reduced upon inhibition of PTP1B activity (Figure 4D). None of these platelet functions were affected by treatment with a selective inhibitor of the protein tyrosine phosphatase SHP1 (NSC87887) (Figure S1-III and Figure S2), demonstrating that specific protein tyrosine phosphatase activity is required for platelet functionality. 

### 3.5. 3-BP Decreases the Capacity of Colorectal Cancer Cell Lines to Activate Platelets

**Figure 5:**
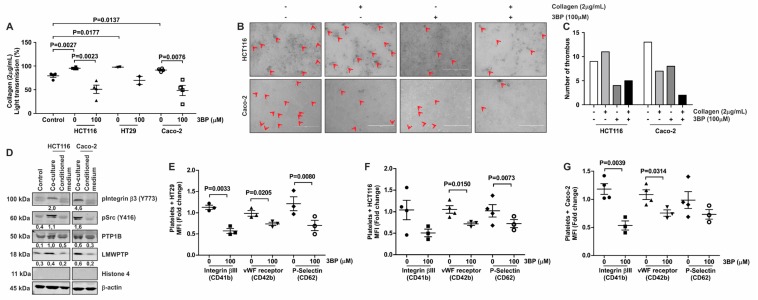
Tumor-cell induced aggregation, thrombus formation and activation are decreased by 3-BP. (**A**) Platelets were pre-incubated with CRC cells (1.5 × 10^4^ cells) and 3-BP (100 μM) for 5 min, and subsequently stimulated using collagen (2 μg/mL). Aggregation was measured for 10 min. Platelets stimulated with collagen only served as controls for the experiment. Each data point (special shapes) corresponds to an individual experiment and indicates the data for platelets marked with: Circle—only platelets, Square—with HCT116, Up-triangle—with HCT116 and 3BP, Down-triangle—with HT29, Diamond—with HT29 and 3BP, Hollow circle—with Caco-2, Hollow Square—with Caco-2 and 3BP. (**B**) Co-cultures of tumor cells and platelets were visualized by microscopy. Platelet aggregation as assessed by microscopy is indicated with arrows and quantified (**C**). (**D**) Platelets from co-culture with CRC cells (HCT116 and Caco-2) were lysed and loaded for western blot analysis of platelet activation markers and phosphatases. β-actin served as loading control. Densitometric values, corrected for loading control, are indicated. A representative blot of at least 2 independent experiments is shown. (**E**–**G**) Platelets co-cultured with HT29 (**E**), HCT116 (**F**) or Caco-2 (**G**) cells in the absence or presence of 3-BP were subsequently subjected to FACS analysis to determine cell surface expression of vWF-receptor (CD41-FITC), Integrinβ3 (CD42-PE) and P-Selectin (CD62-APC). Each data point (special shapes) corresponds to an individual experiment and indicates the data as: Circle—Integrinβ3 without 3BP, Square—Integrinβ3 with 3BP, Up-triangle—vWF-receptor without 3BP, Down-triangle—vWF-receptor with 3BP, Diamond—P-Selectin without 3BP, Hollow circle—P-Selectin with 3BP.

Since the risk of VTE in cancer patients is in part mediated through platelet activation by tumor cells, we investigated the behavior of platelets using co-culture with colorectal cancer cells (HCT116, HT29, and Caco-2) as a model system. As shown in Figure 5A and Figure S1-IV, CRC cells were able to increase collagen-dependent platelet aggregation. Furthermore, treatment of co-cultures with 3-BP significantly reduced both platelet aggregation as determined by lumi-aggrogometry (Figure 5A, Figure S1-IV) and platelet aggregation as suggested by limited counting of aggregates using microscopy (Figure 5B,C). An increased presence of LMWPTP and PTP1B was observed in platelets co-cultured with either HCT116 or Caco-2 cells, with a concomitant upregulation of Integrinβ3 and Src phosphorylation. This effect was mediated through cell-cell contact, as conditioned medium from tumor cells did not elicit the same effect (Figure 5B). As a validation of our findings, we assessed the cell surface expression of platelet activation markers (vWF-receptor, integrin β3 and P-Selectin) in the presence of tumor cells, and showed a significant reduction of these markers upon treatment of co-cultures with 3-BP (Figure 5D–F). Taken together, these data demonstrate that platelet activity can be directly modulated through the physical contact with cancer cells, and show the potential of 3-BP to disturb this cancer cell-platelet interaction.

### 3.6. Hyperactivity of Platelets from Gastrointestinal Cancer Patients Is Reduced upon Treatment with 3-BP

**Figure 6:**
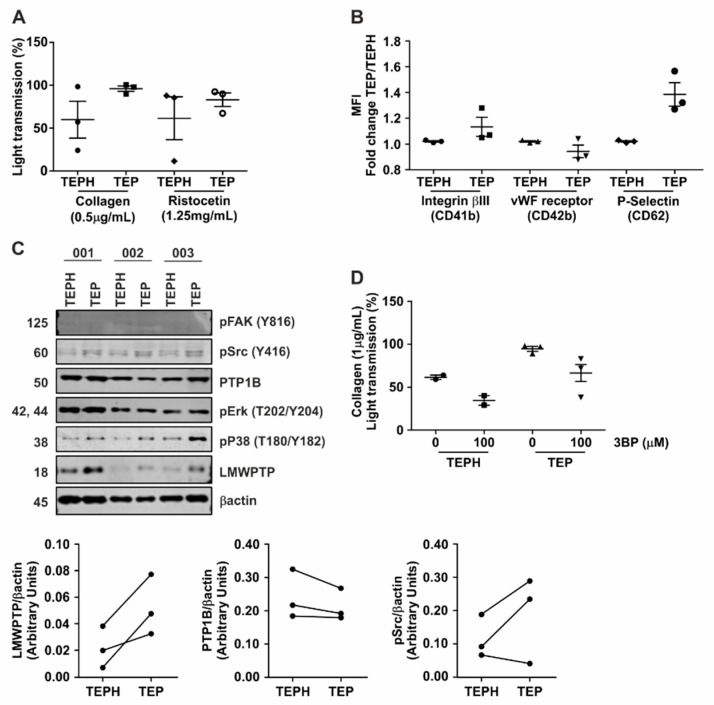
Platelets from patients with gastrointestinal cancer show hyperaggregation which can be inhibited by 3-BP. (**A**) Platelets from 3 gastrointestinal (GI) cancer patients (TEP) and 3 healthy controls (TEPH) were stimulated with either collagen (0.5 μg/mL) or ristocetin (1.25 mg/mL) and the aggregation was measured for 10 min. (**B**) Cell surface expression of vWF-receptor (CD41-FITC), Integrinβ3 (CD42-PE) and P-Selectin (CD62-APC) was investigated on platelets from GI cancer patients (TEP) and healthy controls (TEPH). (**C**) Platelets obtained from GI cancer patients and controls were lysed and subjected to western blot analysis of the indicated (phospho-)proteins. Densitometric analysis of LMWPTP, PTP1B and p-Src expression are shown. (**D**) Aggregation assay using 3-BP (100 μM) as inhibitor of platelet function in 2 healthy controls and 3 GI cancer patients. 3-BP was incubated at pre-test step for 5 min, and after platelets were stimulated using collagen (1 μg/mL).

We performed a small proof-of-concept study to investigate the capacity of 3-BP to decrease platelet aggregation in blood samples from three patients with gastrointestinal cancer. It has previously been described that platelets from cancer patients are more sensitive to in vitro collagen stimulation as compared to healthy controls. While our group is too small to make claims regarding significance as interpersonal variation may exist, we did observe a similar trend (% of light transmission of 95.67 ± 3.167 for patients vs. 59.67 ± 21.40 for controls, Figure 6A, Figure S1-V). However, within the same experimental set-up, ristocetin-triggered aggregation was less different between three patients and three controls, which may suggest that specific molecular dysfunctions of adhesion processes are present in these patients (82.83 ± 7.949 for patients vs. 61.33 ± 24.93 for controls). Accordingly, P-Selectin levels on platelets from cancer patients appeared to be higher as compared to control, although again, we only assessed few patients (Figure 6B). Interestingly, expression of LMWPTP, but not PTP1B, was enhanced in all three patients studied, as compared to the experimental control subjects (Figure 6C). Importantly, 3-BP significantly decreased collagen-stimulated platelet function (Figure 6D, Figure S1-VI). Together, these data suggest that phosphatases are key players in platelet function and aggregation in cancer patients and may be targeted by 3-BP.

## 4. Discussion and Conclusion

Cancer patients, in particular those suffering from gastrointestinal tumors, have a severely increased risk of developing VTE. Although the cause of this increased risk has not yet been fully elucidated, it has been described that tumor cells are capable of enhancing platelet aggregation in a process known as tumor cell-induced platelet aggregation (TCIPA) [37,38]. We investigated the molecular mechanisms associated with platelet hyper-aggregability. In the present study, we confirmed the importance of Src activity for collagen-induced platelet function, and demonstrated that both Src and Integrinβ3 activation are enhanced upon co-culture of platelets with cancer cells. Furthermore, we show here that phosphatases modulating Src activity, i.e., LMWPTP and PTP1B, are present and active in platelets, and that the levels of these phosphatases are enhanced upon co-culture with CRC cells. While it may seem surprising that inhibition of these phosphatases reduces platelet activation which relies so heavily on Src activity, both PTP1B and LMWPTP have previously been shown to be essential for Src activation, which is dependent on the balance between its phosphorylation sites [17,39].

In the present study, we observed differences in phosphatase activation upon stimulation of platelets with different agonists. Unlike collagen, ristocetin was not able to induce LMWPTP activity, suggesting that collagen-induced platelet activation relies more on LMWPTP activity than ristocetin-induced activity. Furthermore, in the cancer patients tested in this study, LMWPTP expression and collagen-induced aggregation, but not ristocetin-induced activity, were increased in comparison to healthy controls. Interestingly, increased LMWPTP mRNA profiles have been identified in platelets from colorectal, pancreatic, breast and hepatobiliary cancer patients [40] which, in light of our current data, suggests that this phosphatase may be related to tumor cell-induced platelet aggregation risk. 

**Figure 7:**
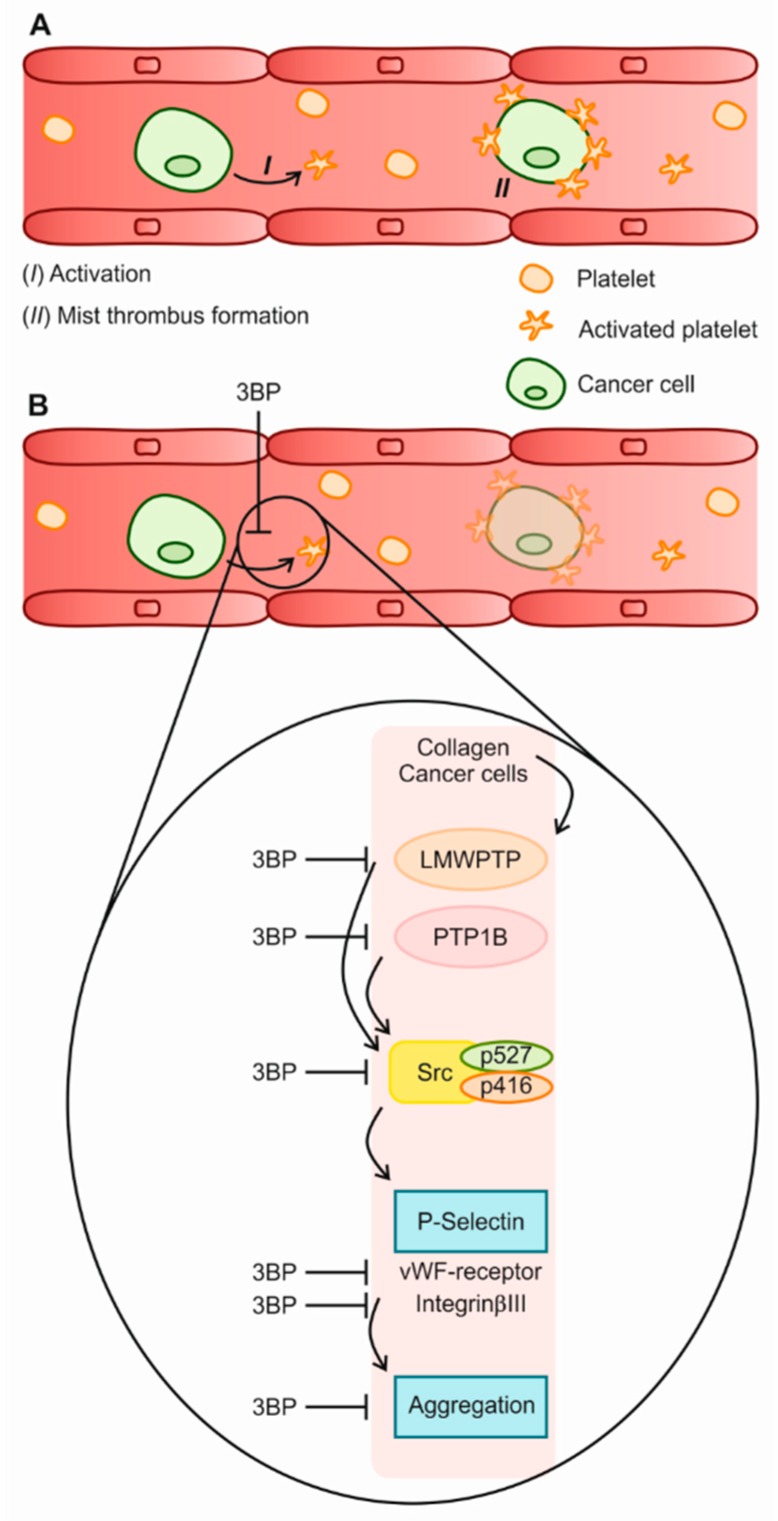
The 3-BP effect on platelet activation. (**A**) Platelets can be activated by collagen and tumor cells. (**B**) In the presence of 3-BP, the modulation of phosphatases plays contributes to a decreased platelet activity. This can culminate in a decreased aggregation, including cancer cell-platelet mist thrombi.

A growing body of evidence point towards an important role of PTPs in platelet biology [41,42]. Recently, it was shown that platelets contain the dual specificity phosphatase DUSP3, and that inhibition of this phosphatase reduces arterial thrombosis in mice [43,44]. A functional role for PTP1B has also been suggested in platelets [45,46]. Here, we show for the first time, that platelet LMWPTP activity is modulated by collagen, but not by ristocetin, and identified this phosphatase as a druggable target for platelet hyperactivity. Importantly, we found that 3-BP diminished the reactivity of platelets from healthy individuals as well as gastrointestinal cancer patients, at least in part through the inhibition of LMWPTP, PTP1B and Src kinaseds. However, the fact that ristocetin-induced aggregation and activation marker expression was not inhibited by 3-BP treatment might suggest that, even though in vitro 3-BP is able to inhibit PTP1B activity, cellular LMWPTP is more affected by 3-BP (Figure 7).

Despite the great progress in the development of cancer treatment protocols, the treatment of several tumors is still a challenge, especially in metastatic cases. The use of antithrombotic treatment as a co-adjuvant strategy in cancer treatment was already suggested in 1982, in particular in its capacity to decrease metastasis [47]. Indeed, it has been described that antithrombotic therapy improves survival in patients with colorectal cancer [48]. We have previously shown that LMWPTP and PTP1B contribute to metastatic potential of (intestinal) cancer cells [24,25]. Here, we demonstrate the important role of these phosphatases in platelet biology. Thus, targeting these phosphatases through 3-BP and its derivatives may provide a potential strategy to reduce both VTE risk and tumor metastasis in GI cancer patients. 

We acknowledge several limitations in this study. Platelet aggregation is a complex, multistep process, requiring many agonists at different time points of the process. Here, we limited our investigations to collagen and ristocetin-induced platelet functions, as previous studies have indicated that in particular collagen-induced platelet aggregation was increased in cancer patients in in vitro experiments [49], and that this correlates well to VTE risk scores [50]. However, in vivo, Tissue Factor may be a more relevant cancer-derived platelet agonist [51], and further studies will have to elucidate the role of phosphatases in the activation of platelets with this and other agonists. Furthermore, these in vitro experiments such as these do not take into account fibrinogen and the plethora of other factors encountered by platelets in vivo, and therefore care should be taken when trying to extrapolate these findings to an in vivo setting. Secondly, platelets are notoriously easily activated in in vitro experiments, and several different isolation and washing protocols to reduce unwanted activation have been published [52,53,54]. The protocol used here was taken from Jankowski et al, as this was the most compatible with downstream analysis of platelets for phosphatase activity assays. While we did not observe unwanted activation in our studies, it is conceivable that other wash protocols may have given slightly different results. Lastly, we only included 3 cancer patients in our proof-of-concept study, and larger studies using more patients and controls matched for age as well as gender, and more different agonists are warranted to further elucidate the role of phosphatases in these settings, and the potential of 3BP to affect platelet function in GI patients. 

## Supplementary Materials

The following are available online at https://www.mdpi.com/2077-0383/8/7/936/s1, Figure S1: Supplemental Figure S1. Representative aggregation graphics from each experiment are combined in one file (pdf). I: Treatment of platelets with collagen/ristocetin in the absence or presence of the Src inhibitor PP2; II: Treatment of platelets with collagen/ristocetin in the absence or presence of 3-BP; Treatment of platelets with collagen in the absence or presence of the PTP1B inhibitor CinnGEL or the SHP inhibitor NSC87887; IV: Treatment of platelets with collagen in the absence or presence of tumor cells (Caco-2, HT29, HCT116) and 3-BP; V: Treatment of patient and control platelets with collagen/ristocetin; VI: Treatment of patient and control platelets with collagen/ristocetin in the absence or presence of 3-BP, Figure S2: Platelet function is not inhibited by selective SHP inhibitor. (A) Aggregation assays were performed for collagen-stimulated platelets after pretreatment with specific SHP inhibitor (NSC87887). (B) Cell surface expression of vWF-receptor (CD41-FITC), Integrinβ3 (CD42-PE) and P-Selectin (CD62-APC) was investigated on collagen-stimulated platelets that were pre-treated with specific SHP inhibitor (NSC87887). Table S1: Western blot antibodies, Table S2: Chemicals, Table S3: Cell line characteristics.
