# Correction: Faria, A.V.S. et al., Targeting Tyrosine Phosphatases by 3-Bromopyruvate Overcomes Hyperactivation of Platelets from Gastrointestinal Cancer Patients. *J. Clin. Med.* 2019, *8*, 936

**DOI:** 10.3390/jcm9082625

**Published:** 2020-08-13

**Authors:** Alessandra V. S. Faria, Sheila S. Andrade, Agnes N. Reijm, Manon C. W. Spaander, Moniek P. M. de Maat, Maikel P. Peppelenbosch, Carmen V. Ferreira-Halder, Gwenny M. Fuhler

**Affiliations:** 1 Department of Gastroenterology and Hepatology, Erasmus University Medical Center Rotterdam, NL-3000 CA Rotterdam, The Netherlands; alessandravsfaria@gmail.com (A.V.S.F.); a.reijm@erasmusmc.nl (A.N.R.); v.spaander@erasmusmc.nl (M.C.W.S.); m.peppelenbosch@erasmusmc.nl (M.P.P.); 2 Department of Biochemistry and Tissue Biology, University of Campinas, UNICAMP, Campinas, SP 13083-862, Brazil; 3 PlateInnove Biotechnology, Piracicaba, SP 13414-018, Brazil; sheilasa@gmail.com; 4 Department of Hematology, Erasmus University Medical Center Rotterdam, NL-3000 CA Rotterdam, The Netherlands; m.demaat@erasmusmc.nl

The authors wish to make the following correction to their paper [1], published on 28 June 2019 in *Journal of Clinical Medicine*. Figure 1A in the published version is incorrect due to the authors’ carelessness. The authors sincerely apologize for this mistake to all the readers. The journal office has checked the review history and found all drafts of the article used the correct picture for Figure 1A apart from the final publication version. Thus, this correction does not alter the interpretation or conclusions drawn from the present study.

Figure 1 should be replaced with the following, correct picture:

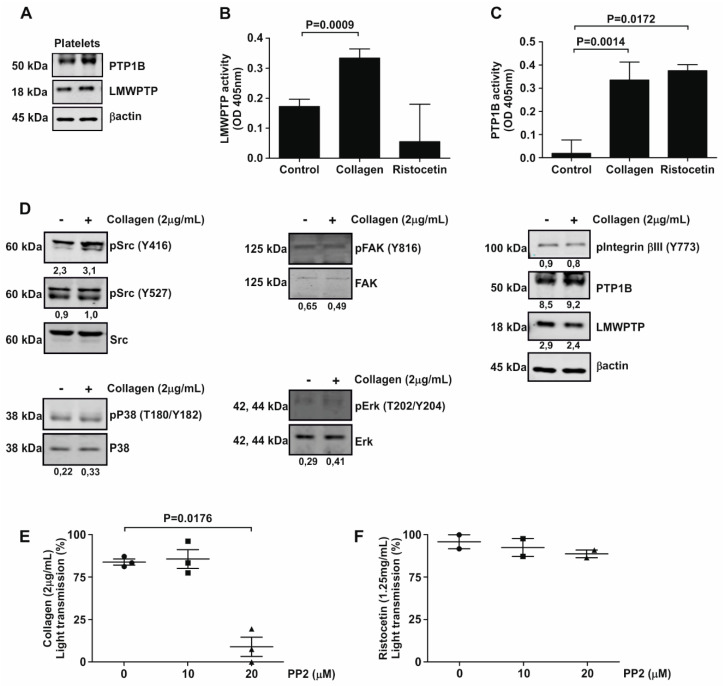



The published version will be updated online on the article webpage, with a reference to this correction.

## References

[1] Faria A.V.S., Andrade S.S., Reijm A.N., Spaander M.C.W., de Maat M.P.M., Peppelenbosch M.P., Ferreira-Halder C.V., Fuhler G.M. (2019). Targeting Tyrosine Phosphatases by 3-Bromopyruvate Overcomes Hyperactivation of Platelets from Gastrointestinal Cancer Patients. J. Clin. Med..

