# Supplementary material for: Targeting Tyrosine Phosphatases by 3-Bromopyruvate Overcomes Hyperactivation of Platelets from Gastrointestinal Cancer Patients
Source: J Clin Med. 2019 Jun 28;8(7):936. doi: 10.3390/jcm8070936 (PMC6678874; doi:10.3390/jcm8070936)
Supplement: Supplementary file 1 [file jcm-08-00936-s001.zip › jcm-517891-supplementary-approved/Legends for supplemental figures.docx]

**Legends for supplemental figures**

**Supplemental Figure S1. Representative aggregation graphics from each experiment are combined in one file (pdf).** I: Treatment of platelets with collagen/ristocetin in the absence or presence of the Src inhibitor PP2; II: Treatment of platelets with collagen/ristocetin in the absence or presence of 3-BP; Treatment of platelets with collagen in the absence or presence of the PTP1B inhibitor CinnGEL or the SHP inhibitor NSC87887; IV: Treatment of platelets with collagen in the absence or presence of tumor cells (Caco-2, HT29, HCT116) and 3-BP; V: Treatment of patient and control platelets with collagen/ristocetin; VI: Treatment of patient and control platelets with collagen/ristocetin in the absence or presence of 3-BP.

**Figure 2. Platelet function is not inhibited by selective SHP inhibitor.** (A) Aggregation assays were performed for collagen-stimulated platelets after pretreatment with specific SHP inhibitor (NSC87887). (B) Cell surface expression of vWF-receptor (CD41-FITC), Integrinβ3 (CD42-PE) and P-Selectin (CD62-APC) was investigated on collagen-stimulated platelets that were pre-treated with specific SHP inhibitor (NSC87887).
