# Supplementary figures and images for: Targeting Tyrosine Phosphatases by 3-Bromopyruvate Overcomes Hyperactivation of Platelets from Gastrointestinal Cancer Patients
Source: J Clin Med. 2019 Jun 28;8(7):936. doi: 10.3390/jcm8070936 (PMC6678874; doi:10.3390/jcm8070936)

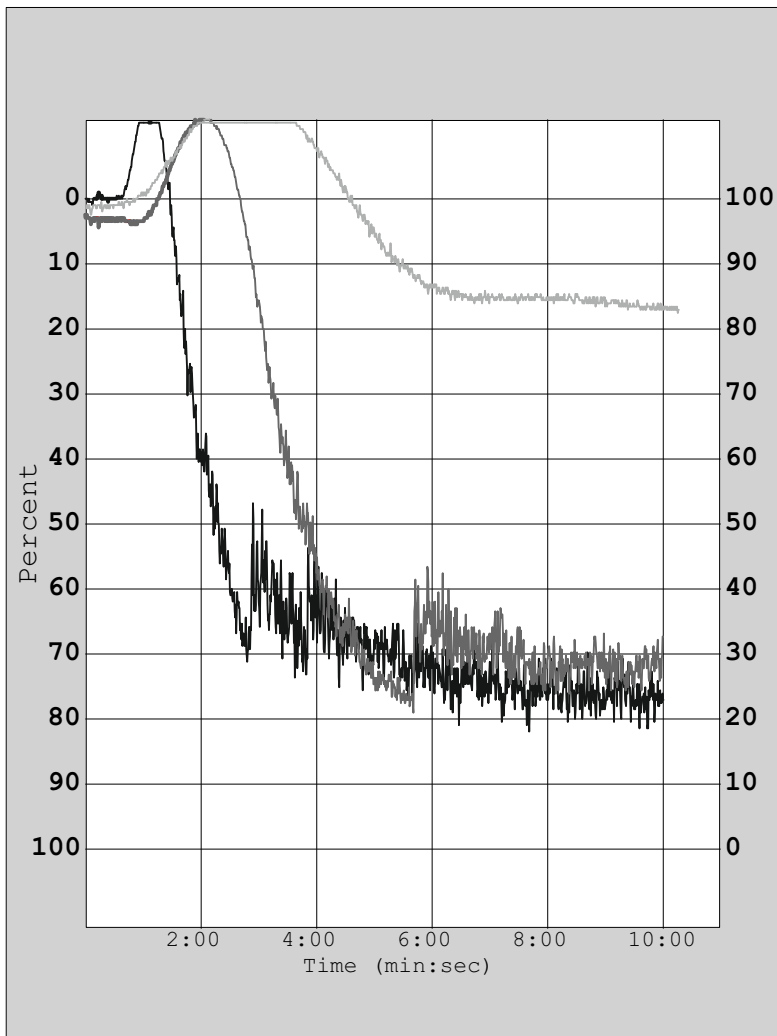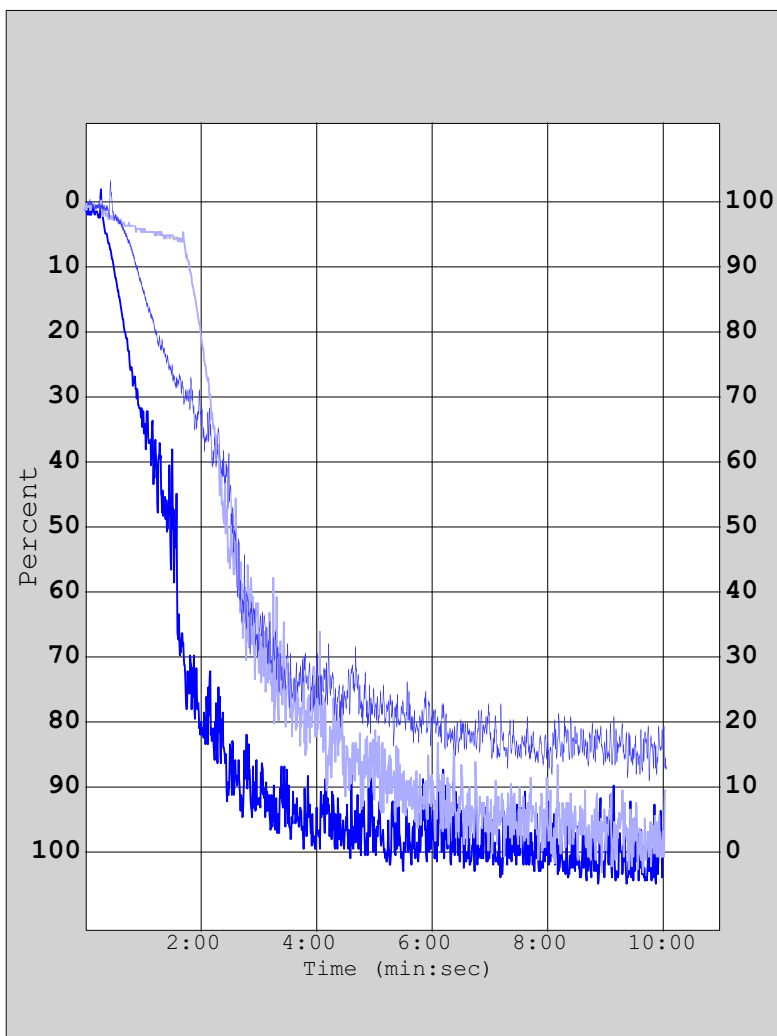

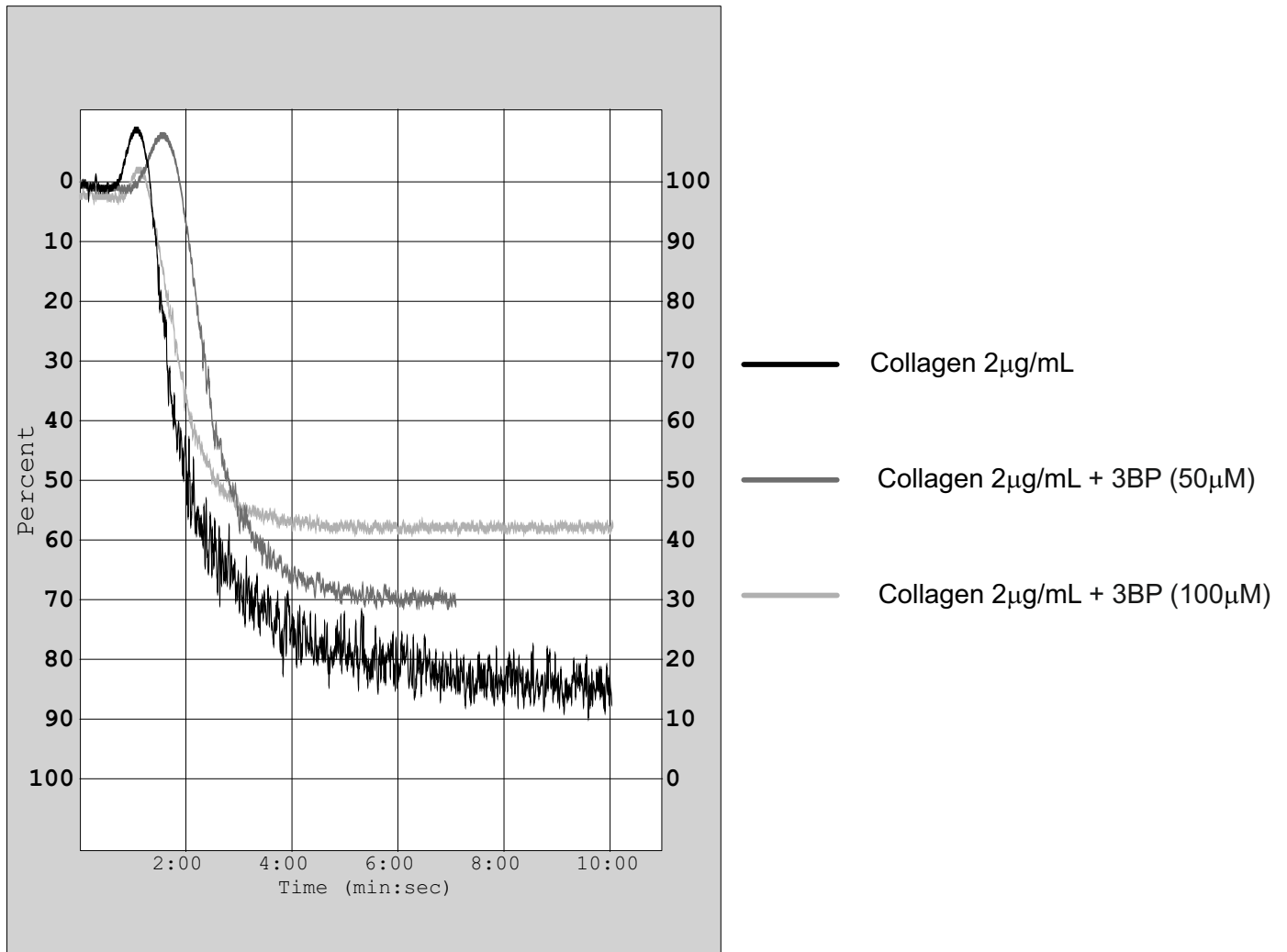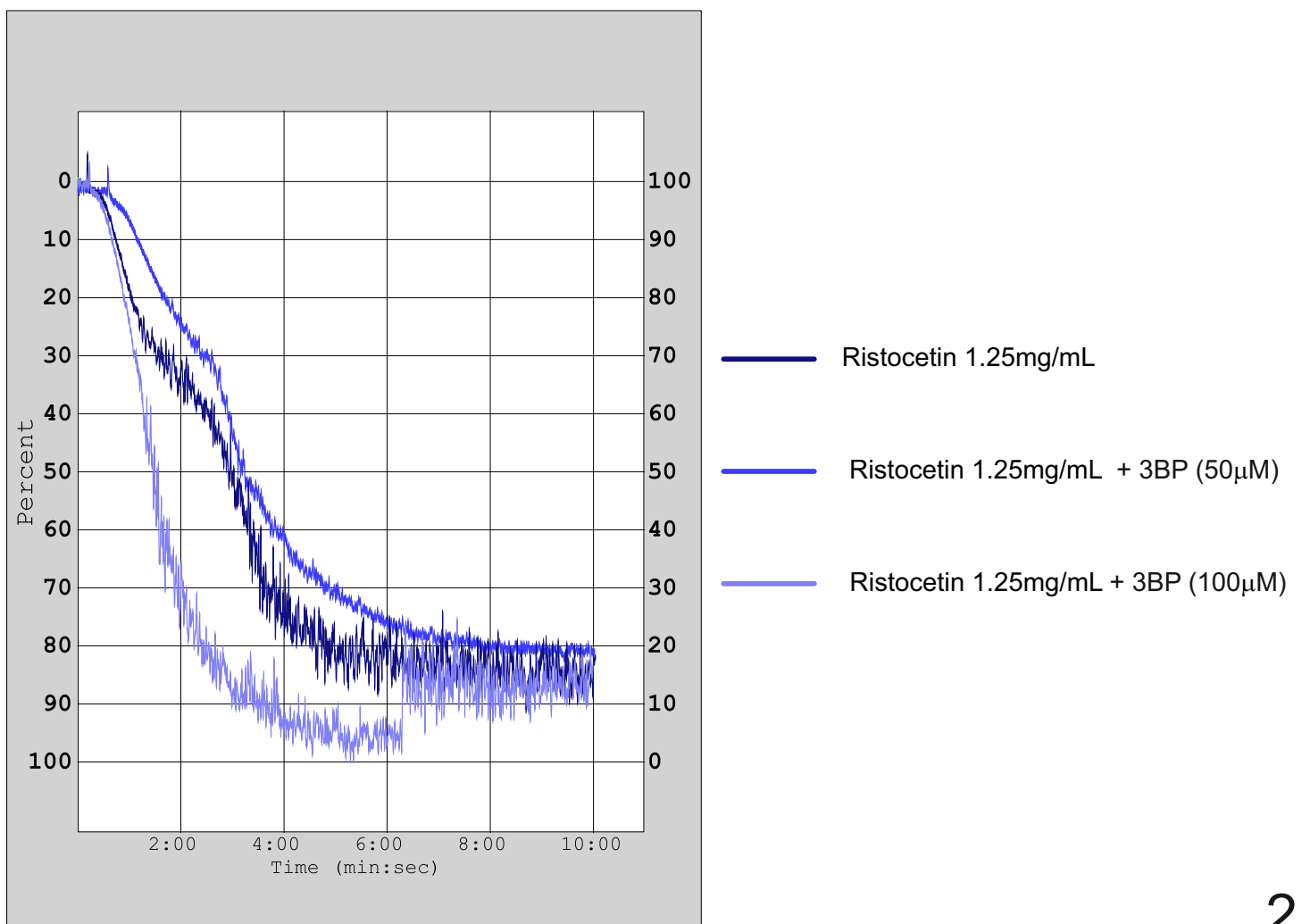

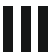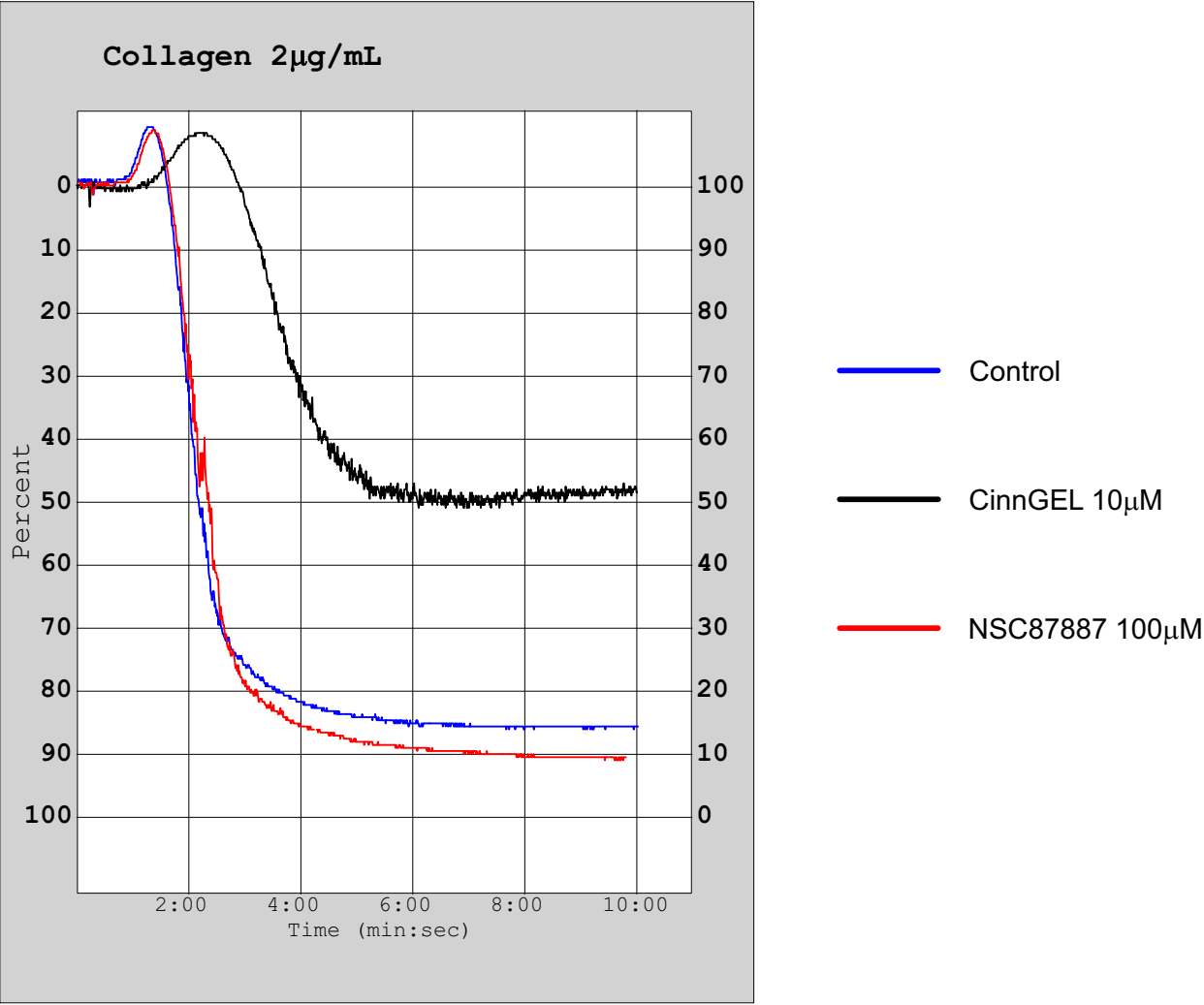

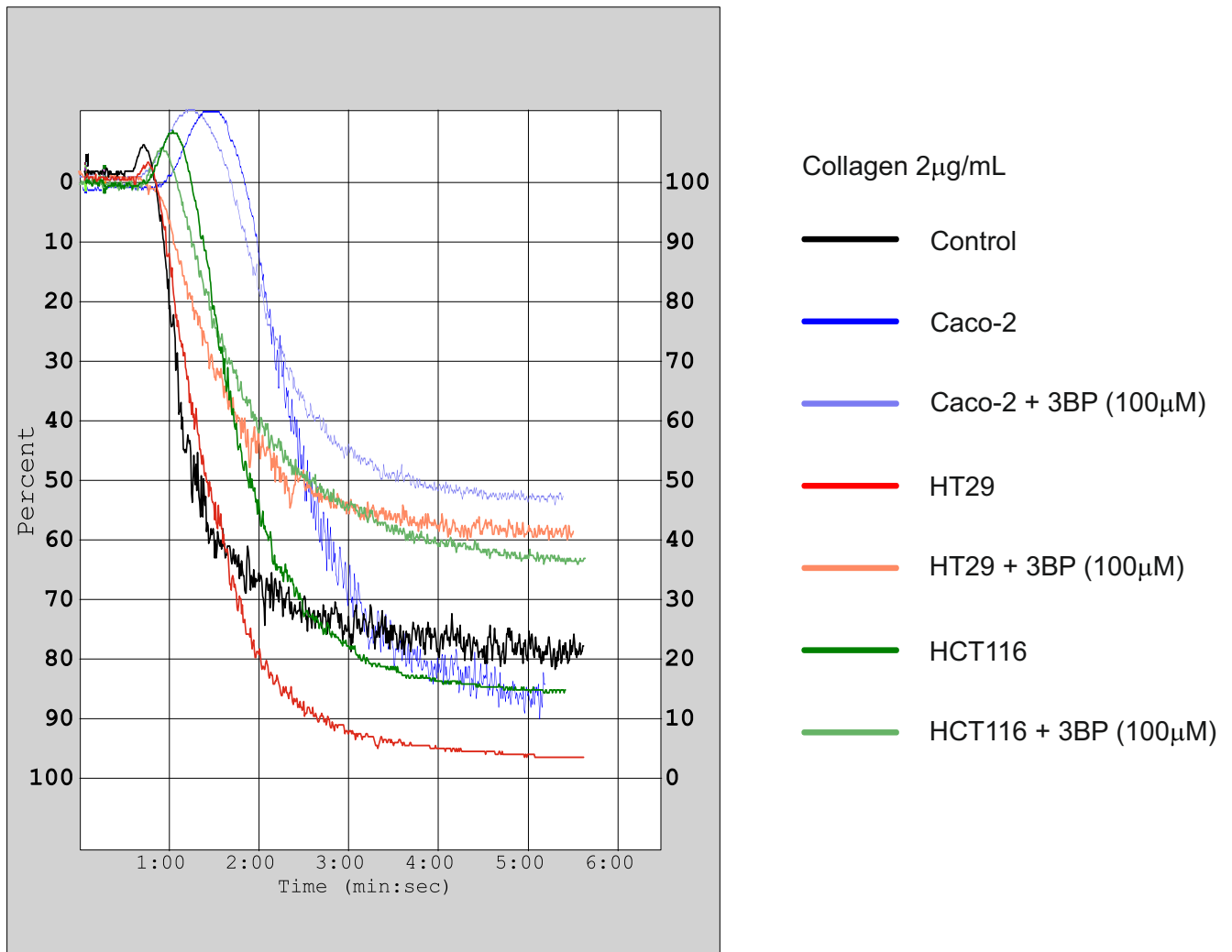

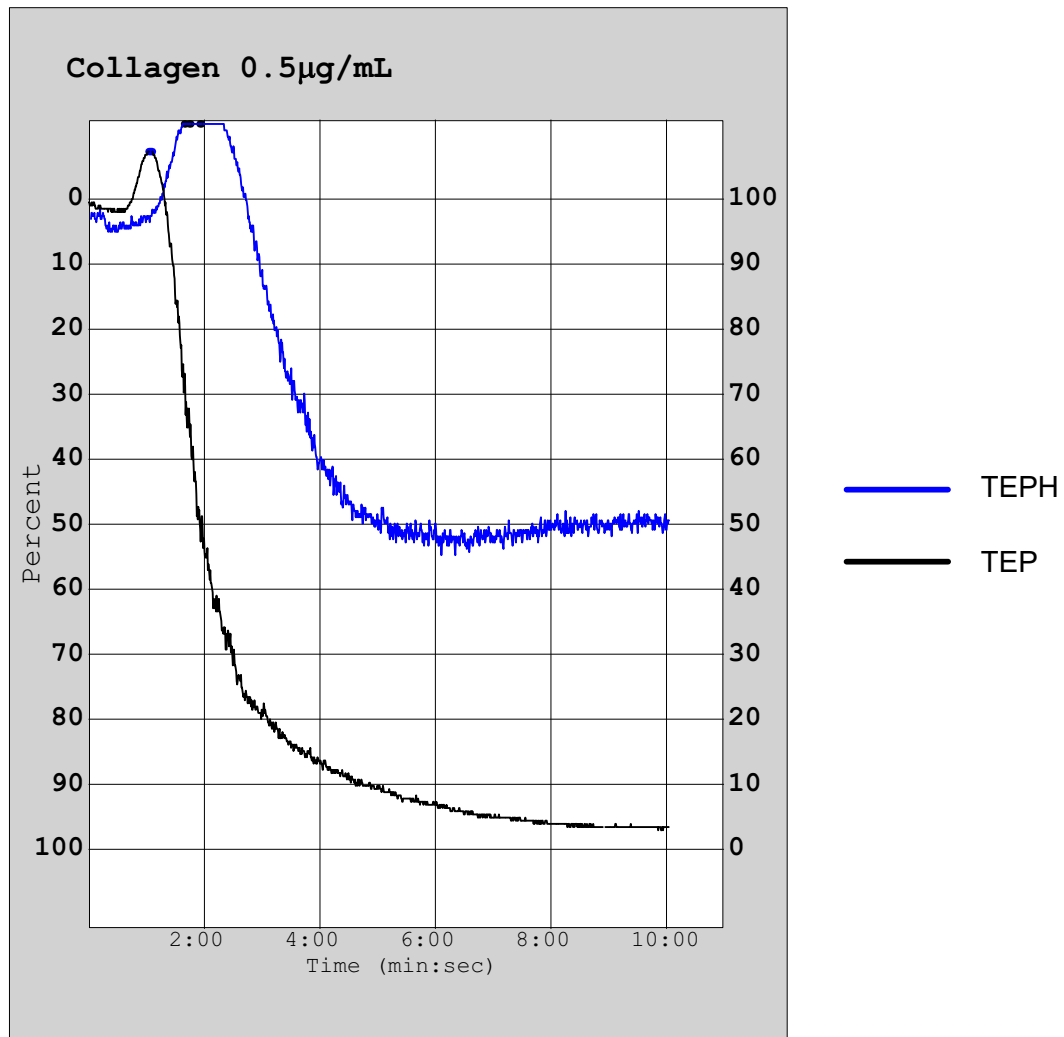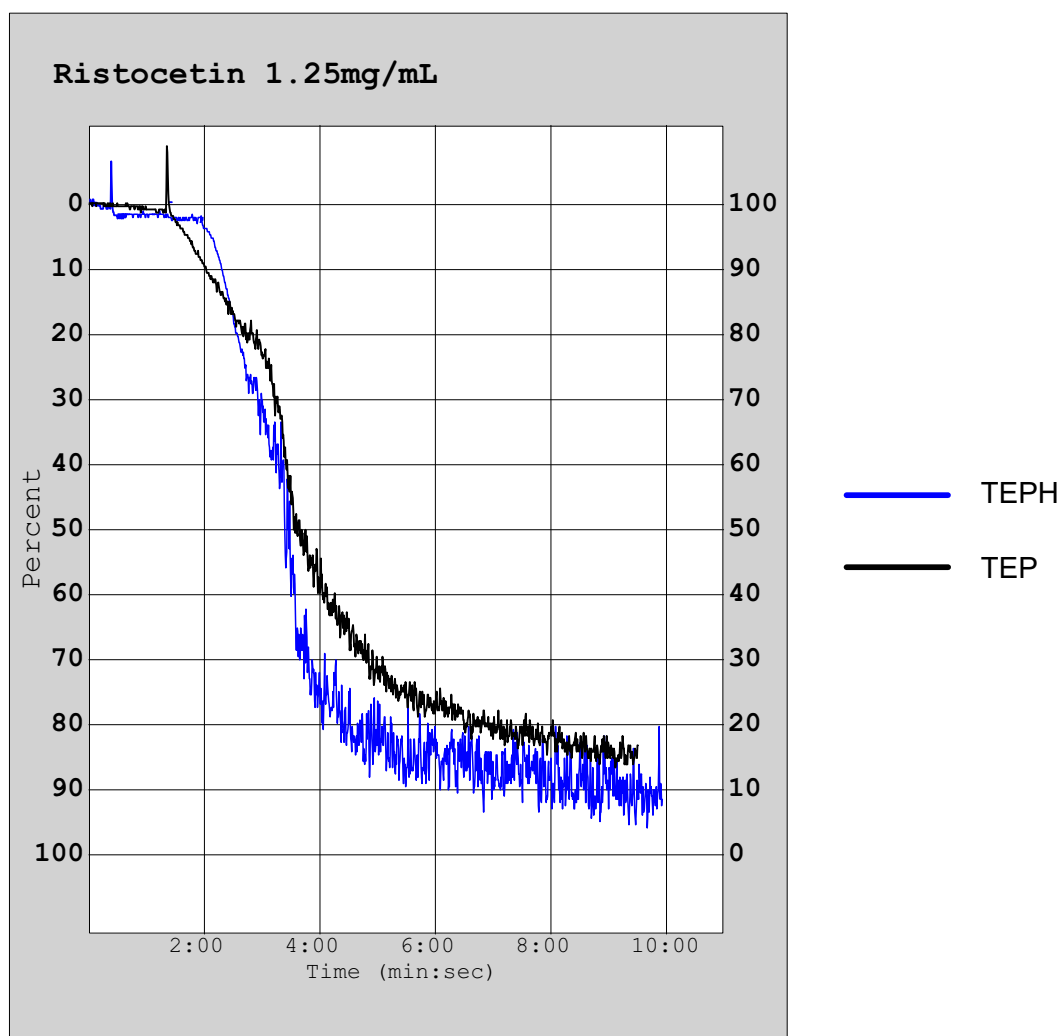

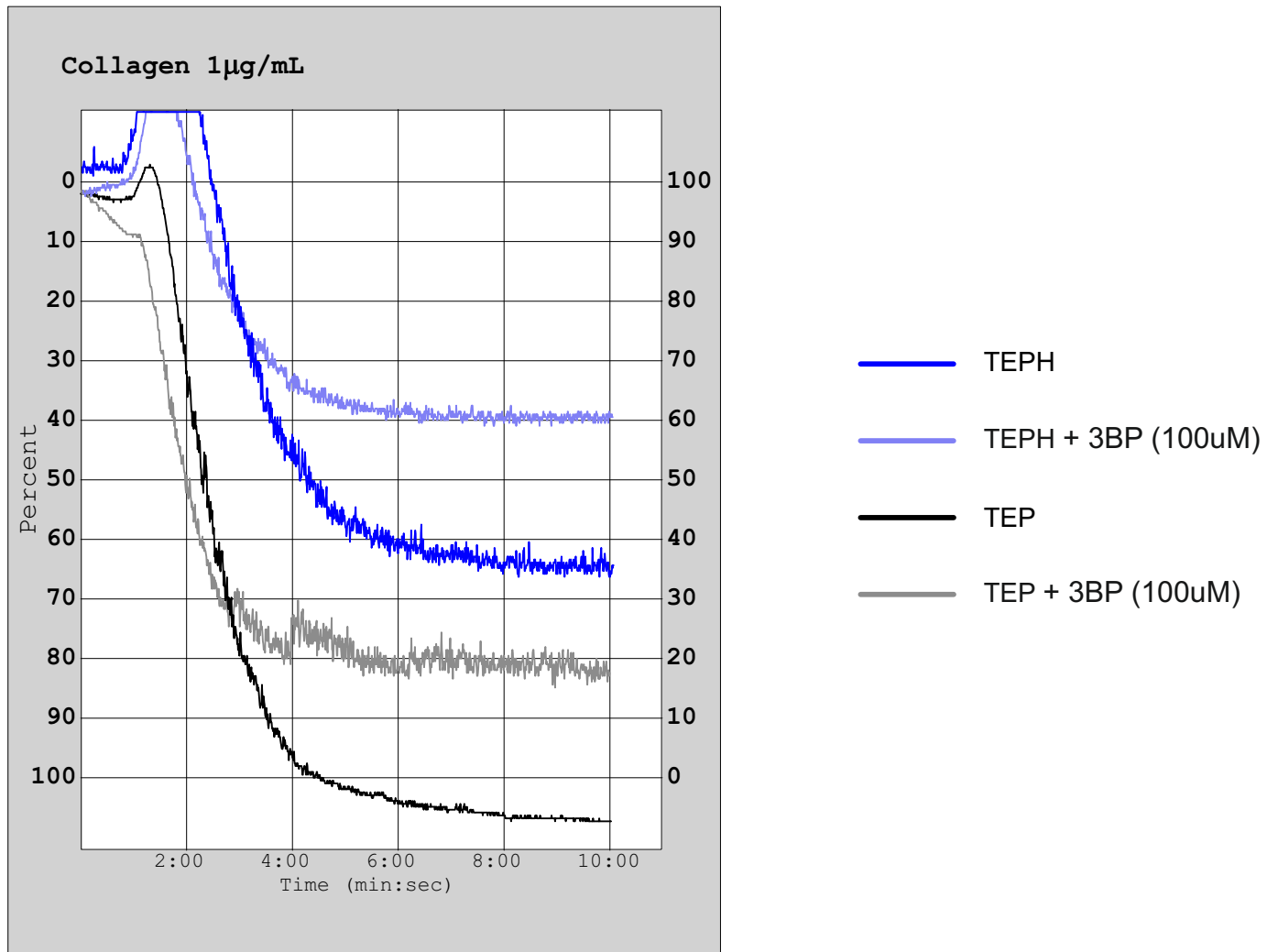

Supplement: Supplementary file 1 [file jcm-08-00936-s001.zip › jcm-517891-supplementary-approved/figure S1.pdf]
