# Supplementary material for: Targeting Tyrosine Phosphatases by 3-Bromopyruvate Overcomes Hyperactivation of Platelets from Gastrointestinal Cancer Patients
Source: J Clin Med. 2019 Jun 28;8(7):936. doi: 10.3390/jcm8070936 (PMC6678874; doi:10.3390/jcm8070936)
Supplement: Supplementary file 1 [file jcm-08-00936-s001.zip › jcm-517891-supplementary-approved/jcm-517891-Table S.doc]

**Supplemental information**

**Supplemental tables**

Table 1. Western blot antibodies

| Antibody | Company | Code |
| --- | --- | --- |
| Acp1 α/β | Santa Cruz Biotechnologies | sc-100343 |
| PTP1B | Santa Cruz Biotechnologies | sc-14021 |
| βactin | Santa Cruz Biotechnologies | sc-47778 |
| Phospho-Src family (Tyr416) | Cell Signaling | 2101 |
| Phospho-Src (Tyr572) | Cell Signaling | 2105 |
| Src | Cell Signaling | 2123 |
| Phospho-FAK (Tyr925) | SignalWay Antibodies | 11123-2 |
| FAK | Cell Signaling | 3285S |
| Phospho-Integrin βIII (Tyr773) | SignalWay Antibodies | 11060-1 |
| Phospho-Erk (T202/Y204) | Cell Signaling | 4370 |
| Erk | Cell Signaling | 4695 (137F5) |
| Phospho-p38 (T180/Y182) | Cell Signaling | 4511 |
| p38 | Cell Signaling | 9228 |
| Caspase-3 | Cell Signaling | 9662 |
| Cleaved Caspase-8 | Cell Signaling | 9496 |
| Bax | Cell Signaling | 2772 |
| Bcl-2 | Cell Signaling | 2876 |

Table 2. Chemicals

| Reagent | Company | Code |
| --- | --- | --- |
| 3-bromopyruvate | Sigma Aldrich | 16490-10 |
| CinnGel | Santa Cruz | 205633 |
| NSC87887 | Merck Millipore | 565851 |
| Collagen | Chronolog | 385 |
| Ristocetin | Chronolog | 396 |

Table 3. Cell line characteristics, as described in [1-9].

| **Cell line** | **HCT116** | **HT29** | **Caco-2** |
| --- | --- | --- | --- |
| **Patient** | 48-Year-old male | 44-Year-old female | 72-Year-old male |
| **Organ** | Colon ascendens | Colon | Colon |
| **Disease** | colorectal carcinoma | colorectal adenocarcinoma | colorectal adenocarcinoma |
| **Stage** | Dukes’ D | Dukes’ C |  |
| **Derived from** | primary tumor | primary tumor |  |
| **MSI status** | MSI | MSS | MSS |
| **CIMP panel 1** | + | + | + |
| **CIMP panel 2** | + | + | - |
| **CIN** | - | + | + |
| **KRAS** | G13D | WT | WT |
| **BRAF** | WT | V600E | WT |
| **PIK3CA** | H1047R | P449T | WT |
| **PTEN** | WT | WT | WT |
| **TP53** | WT | R273H | E204X |

Abbreviations: CIN, chromosomal instability pathway; MSI, microsatellite instability; MSS, microsatellite stable; CIMP, CpG island methylator phenotype

1. Ahmed D, Eide PW, Eilertsen IA, Danielsen SA, Eknæs M, Hektoen M, Lind GE, Lothe RA. (2013). Epigenetic and genetic features of 24 colon cancer cell lines. Oncogenesis. 16, 71.
2. Brattain MG, Brattain DE, Fine WD, Khaled FM, Marks ME, Kimball PM, Arcolano LA, Danbury BH. (1981). Initiation and characterization of cultures of human colonic carcinoma with different biological characteristics utilizing feeder layers of confluent fibroblasts. Oncodev Biol Med. 2(5):355-66.
3. Brattain MG, Fine WD, Khaled FM, Thompson J, Brattain DE. (1981). Heterogeneity of malignant cells from a human colonic carcinoma. Cancer Res. 41(5):1751-6.
4. Eshleman JR, Lang EZ, Bowerfind GK, Parsons R, Vogelstein B, Willson JK, Veigl ML, Sedwick WD, Markowitz SD. (1995). Increased mutation rate at the hprt locus accompanies microsatellite instability in colon cancer. Oncogene. 10(1):33-7.
5. Fogh J, Wright WC, Loveless JD. (1977). Absence of HeLa Cell Contamination in 169 Cell Lines Derived From Human Tumors. JNCI: Journal of the National Cancer Institute, 58(2), 209–214. doi:10.1093/jnci/58.2.209
6. Griffin C, Karnik A, McNulty J, Pandey S. (2011). Pancratistatin selectively targets cancer cell mitochondria and reduces growth of human colon tumor xenografts. Mol. Cancer Ther. 10; 57-68.
7. Liu Y, Bodmer WF. (2006). Analysis of P53 mutations and their expression in 56 colorectal cancer cell lines. Proc Natl Acad Sci U S A. 103; 976-81.
8. Mouradov D, Sloggett C, Jorissen RN, Love CG, Li S, Burgess AW, Arango D, Strausberg RL, Buchanan D, Wormald S, O'Connor L, Wilding JL, Bicknell D, Tomlinson IP, Bodmer WF, Mariadason JM, Sieber OM. (2014). Colorectal cancer cell lines are representative models of the main molecular subtypes of primary cancer. Cancer Res. 74, 3238-47.
9. Takawa M, Masuda K, Kunizaki M, Daigo Y, Takagi K, Iwai Y, Cho HS, Toyokawa G, Yamane Y, Maejima K, Field HI, Kobayashi T, Akasu T, Sugiyama M, Tsuchiya E, Atomi Y, Ponder BA, Nakamura Y, Hamamoto R. (2011). Validation of the histone methyltransferase EZH2 as a therapeutic target for various types of human cancer and as a prognostic marker. Cancer Sci. 102; 1298-305.
